# Effects of photosynthetic models on the calculation results of photosynthetic response parameters in young *Larix principis-rupprechtii* Mayr. plantation

**DOI:** 10.1371/journal.pone.0261683

**Published:** 2021-12-31

**Authors:** Xuemei Ma, Qiang Liu, Zhidong Zhang, Zewen Zhang, Zeyu Zhou, Yu Jiang, Xuanrui Huang

**Affiliations:** 1 College of Forestry, Hebei Agricultural University, Baoding Hebei, China; 2 Anyang Institute of Technology Anyang Henan, Anyang, China; Tennessee State University, UNITED STATES

## Abstract

Accurately predicting the crown photosynthesis of trees is necessary for better understanding the C circle in terrestrial ecosystem. However, modeling crown for individual tree is still challenging with the complex crown structure and changeable environmental conditions. This study was conducted to explore model in modeling the photosynthesis light response curve of the tree crown of young *Larix principis-rupprechtii* Mayr. Plantation. The rectangular hyperbolic model (RHM), non-rectangular hyperbolic model (NRHM), exponential model (EM) and modified rectangular hyperbolic model (MRHM) were used to model the photosynthetic light response curves. The fitting accuracy of these models was tested by comparing determinants coefficients (*R*^2^), mean square errors (*MSE*) and Akaike information criterion (*AIC*). The results showed that the mean value of *R*^2^ of MRHM (*R*^*2*^ = 0.9687) was the highest, whereas *MSE* value (*MSE* = 0.0748) and *AIC* value (*AIC* = -39.21) were the lowest. The order of fitting accuracy of the four models for *P*_n_-*PAR* response curve was as follows: MRHM > EM > NRHM > RHM. In addition, the light saturation point (*LSP*) obtained by MRHM was slightly lower than the observed values, whereas the maximum net photosynthetic rates (*P*_max_) modeled by the four models were close to the measured values. Therefore, MRHM was superior to other three models in describing the photosynthetic response curve, the accurate values were that the quantum efficiency (*α*), maximum net photosynthetic rate (*P*_max_), light saturation point (*LSP*), light compensation point (*LCP*) and respiration rate (*R*_d_) were 0.06, 6.06 μmol·m^-2^s^-1^, 802.68 μmol·m^-2^s^-1^, 10.76 μmol·m^-2^s^-1^ and 0.60 μmol·m^-2^s^-1^. Moreover, the photosynthetic response parameters values among different layers were also significant. Our findings have critical implications for parameter calibration of photosynthetic models and thus robust prediction of photosynthetic response in forests.

## Introduction

As the largest carbon flux in global carbon (C) cycling, photosynthesis can assimilate CO_2_ from the atmosphere and thus dedicating to climate change mitigation. It plays a crucial role in the material cycle and energy flow of forest ecosystems [1–3]. In addition to the stage of leaf development and genetic constitution of plant, photosynthesis is also strongly affected by surrounding environmental conditions (e.g., light, leaf, CO_2_ concentration, humidity and temperature etc.), during which intensity of light and its availability particularly determine the amount of C assimilated by photosynthesis [4–7]. For the trees, the canopy is the most direct part for photosynthesis to response to incoming solar irradiance. Therefore, better understanding the mechanism of crown leaf photosynthesis response to light availability is thus critical for maintaining forest productivity and management, in particular for dynamic simulation growth models and in parameterization of crown photosynthesis.

Light response curves (*P*n-*PAR* curves) describe the relationship between net photosynthetic rate (*P*_n_) and photosynthetically active radiation (*PAR*), and provide information about the photosynthetic efficiency of plants (e.g. quantum yield, the maximum photosynthetic capacity, light compensation point and leaf radiation use efficiency of leaves) [8–10]. The simulation of *P*n-*PAR* curves are becoming increasingly important to study the photosynthetic response process of plants to the environment, and analyze the primary productivity of vegetation and forests [1, 11–13]. A series of photo-physiological core parameters, such as maximum net photosynthetic rate (*P*_max_), apparent quantum yield (*AQY*), light-saturation point (*LSP*), light-compensation point (*LCP*), and dark respiration rate (*R*_d_), can be used to assess the canopy photosynthetic rate and capacity of plants in different growth stage. To date, in order to investigate the response of net photosynthetic rate (*P*_n_) to light intensity of different plants, many models, including the rectangular hyperbola model (RHM) [14, 15], the nonrectangular hyperbola model (NRHM) [16, 17], the exponential model (EM) [18, 19], and the modified rectangular hyperbola model (MRHM) [20], have been widely applied in modeling the photosynthetic light-response curve (*P*_n_-*PAR* curve) [1, 12]. However, RHM, NRHM, and EM are very complex, some photo- and biochemical parameters (such as *P*_max_ and *LSP*) are subject to environmental conditions and cannot be calculated directly using these models when light intensity are above zero [21–24], and the fitted values of photosynthetic parameters were significantly different from the measured ones [20, 25]. In the contrary, owing to the addition of two adjusting factors (β and γ) into this model, which made the model highly advantageous in fitting the photo-inhibition and light saturation stages [20, 26], the MRHM can directly produce *P*_max_ and *LSP*, and overcome the limitation of above three models, the accuracy were higher and the results were suitable for fitting *P*_n_-*PAR* curve and photosynthetic parameters under various environmental conditions [20, 24, 26, 27], it has been successfully applied in simulating light-response curves of many plants, such as *Keteleeria calcarea* [28], *Pinu stabulaeformis Carr*. [29], *Pinus koraiensis* [30], *Betula utilis* [31].

*Larix principis-rupprechtii* Mayr (*Larch*)., one of the main species of the total area of all plantations in Northern China, plays an important role in wood production, biodiversity protection, and forest ecological construction, due to its advantages of fast growth, strong adaptability, and high economic value. To the best of our knowledge, little attention has been paid to the application of a variety of dynamic crown photosynthetic light-response models in *Larch*, and the fitting effect and differences of light responses by these models remains unclear. Therefore, the determinant coefficients (*R*^2^), mean square error (*MSE*), and Akaike information criterion (*AIC*) were used to evaluated the performance of four types of light-response models(such as RHM, NRHM, EM, and MRHM) in 16-years-old *Larch*. Planation. The objectives of the study were to select an optimal *P*_*n*_*-PAR* curve model for fitting the *P*_n_*-PAR* curves of *Larch*, and to explore the relationships between the parameters of the optimal *P*_n_-*PAR* curve model and leaf vertical positions. The results are helpful to further explore the spatial heterogeneity of carbon sequestration capacity of *Larch* needles in canopy, and provide a basis for accurately estimating photosynthetic physiological characteristics and the productivity of its plantation.

## Materials and methods

### Ethics statement

#### Conflicts of interest

The authors declare no conflict of interest.

#### Ethical approval

The authors declare that this article does not contain any research with humans or animal subjects.

### Site description and sample tree selection

**Fig 1:**
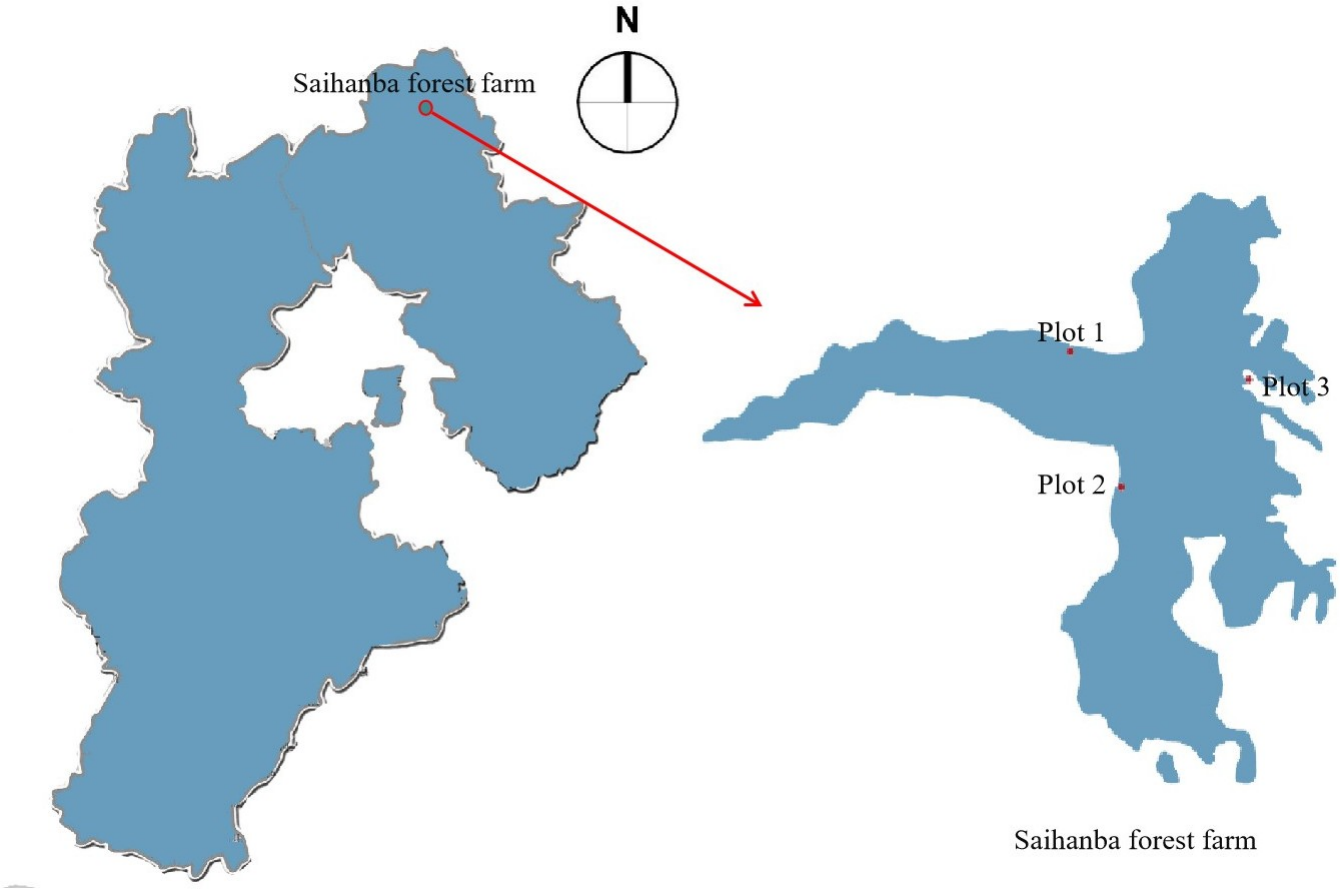
Study location and field experiment of *L*. *principis-rupprechtii* in Hebei Province, China.

The experiments were conducted in the field at the the scientific research base of the State Forestry and Grassland Bureau established by Hebei Agricultural University, and the practice Base for postgraduate training of Forestry Master’s Degree in Hebei Agricultural University. which is located in Saihanba Forest Farm of Weichang County of Hebei Province in the northern China (Fig 1, Table 1). The farm was mainly composed with *Larixprincipis-rupprechtii*, *Populusdavidiana*, *Betula platyphylla*, and *Quercus mongolica*. The total forest coverage is approximately 82.6%, including 72.6% plantation.

**Table 1:** Description of sample site used in the experiment.

Sample site	Latitude (N)	Longitude (E)	Elevation (m)	Climate	Annual mean temperature (°C)	Annual precipitation (mm)	Annual evaporation (mm)	Sunshine hours (h_s_)	Number of frost-free period (d)	Population size (hm^2^)
Saihanba Forest Farm	42°02′-42°36′	116°51′-117°39	1500–2067	cold temperate semi-arid and semi humid continental monsoon	-1.5 (from -42.8 to 30.9°C)	452.6	1230	2368	60	93333

**Fig 2:**
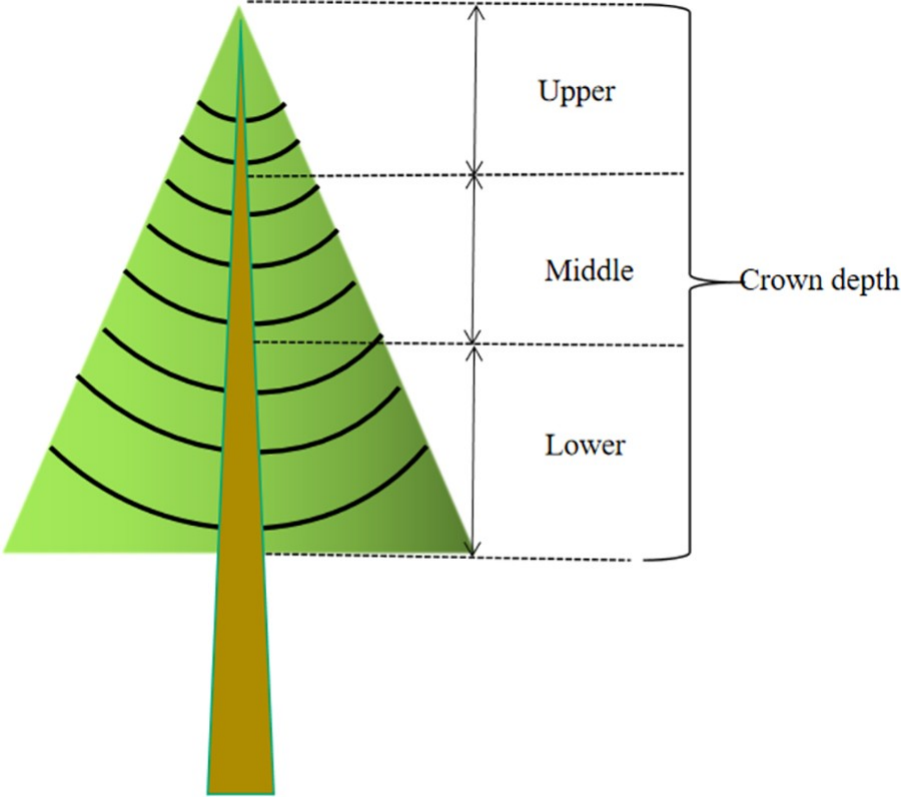
Sketch map of the crown divisions. Upper, Middle and Lower represent three equal divisions of crown depth in the vertical direction.

Three sample plots (20 m width × 30 m length) were set up within 16-year-old *Larch* plantations of the same habitat. The diameter at breast height (DBH, cm) and tree height (H, m) were measured for all trees with the D >5 cm in each plot, and the mean D (Dm) for three plots were calculated independently. Then, three sample trees, whose D values respectively was similar to Dm of the three plots, were selected to represent experimental materials. According to the previous studies, for trees, the upper limit of the *P*_*n*_*-PAR* curves was significantly different within different crown whorls in the vertical direction [32–34]. Thus, we divided the crowns of three sample trees respectively into three vertical layers with the trisection of crown depth (the distance from the top of the tree to the base of its live crown, CD), and each layer was divided into two parts in horizontal direction (sunny and shaded) **(Fig 2)** [35].

### Measurements of the light-response process

The light responses of photosynthesis were measured using a portable photosynthetic gas analysis system (LI-6400, LI-COR, Inc., Lincoln, Nebraska, USA) coupled with a standard red-blue light-emitting diode (LED) radiation source (85% red, emission peak at 655 nm + 15% blue, emission peak at 465 nm) (Li-6400-02B, LI-COR, Inc., Lincoln, NE, USA), photosynthetic active radiations (PAR) intensities were set at thirteen lever of 2000, 1500, 1200, 1000, 800, 600, 400, 200, 150, 100, 50, 25, and 0 μmol (photons) m^-2^s^-1^). Before measuring, the instrument was preheated and calibrated each time, these sample needles were kept for 10–20 min at a CO_2_ concentration of 380 μmol (photons) m^-2^ s^-1^ and a *PAR* value of 1,400 μmol (photons) m^-2^ s^-1^ in the leaf chamber, which reach a steady state around the needles. Then, the sample needles were allowed to equilibrate to 20°C conditions for a minimum time of 2 min and a maximum of 3 min before the data were logged during the measurement of the *P*_n_*-PAR* curves. The measurements (experiments) were conducted from 8:30 a.m. to 16:30 p.m. on a cloud-free periods day, with an air temperature at 24–26°C and a relative humidity at 30–40%, the fixed exposure time for each level of *PAR* was set at 2–3 min, these methods are described previously [32, 33]. The data for the *P*_n_*-PAR* curves were measured once every half month during the growing season (approximately from 15th June to 10th August) in 2018 and 2019

### Photosynthesis-light response curve-fitting model and its parameters

The rectangular hyperbola model, the nonrectangular hyperbola model, the exponential model, and the modified rectangular hyperbola model were used to fit the light-response curves and to estimate photosynthetic parameters. The environmental conditions (CO_2_ concentration, temperature and humidity) are given. The expressions and parameters of the four models were as follows:

The rectangular hyperbola model (RHM) [15, 36] was represented to the following form:

$$P_{n}=\frac{\alpha IP_{max}}{\alpha I+P_{max}}-R_{d}$$ (1)

Where: *P*_n_ represents the net photosynthetic rate (μmol (photon) m^-2^·s^-1^), *a* represents the initial quantum efficiency at low light intensities [22], *P*_max_ represents the maximum net photosynthetic rate (μmol (photon) m^-2^·s^-1^), *R*_d_ represents the respiration rate in the dark (μmol (photon) m^-2^·s^-1^), and *I* represents the *PAR*. *α*, *P*_max_, and *R*_d_ are the main parameters to used describe the characteristics of the *P*_n_*-PAR*. ***Α*** is the initial slope of the *P*_n_*-PAR* when *PAR* is 0–200 μmol·m^-2^·s^-1^, which indicates the plants’ light use efficiency [37–39].

The *P*_max_ and *LSP* cannot be calculated directly using RHM, therefore, *P*_max_ was estimated and calculated by using the nonlinear least squares method under high light intensity [36, 40, 41], *LSP* could be expressed respectively as:

$$P_{\max}=A\times LSP‐R_{d}$$ (2)

where: *A* (*AQE*) represents apparent quantum efficiency; *LSP* represents the light saturation point (μmol (photon) m^-2^·s^-1^); *R*_d_ is as described above. *A* was obtained by fitting the light response data which *PAR* is equal to or less than 200 μmol (photon) m^-2^s^-1^)

$$LSP=(P_{\max}+R_{d})/A$$ (3)

$$LCP=(R_{d}\times P_{\max})/\alpha \times (P_{\max}‐R_{d})$$ (4)

Where: *LCP* represents the light compensation point (μmol (photon) m^-2^·s^-1^); *LSP*, *A*, *P*_max_, *R*_d_ are as described above.

The nonrectangular hyperbola model (NRHM) [42] was represented to the following form:

$$P_{n}=\frac{\alpha I+P_{max}-\sqrt{{\left(\alpha I+P_{max}\right)}^{2}-4\alpha \theta IP_{max}}}{2\theta }-R_{d}$$ (5)

where: *P*_n_ indicates the net photosynthetic rate (μmol (photon) m^-2^·s^-1^); *θ* (0 < *θ* < 1) indicates the convexity (curvilinear angle) (dimensionless); and *α*, *I*, *P*_max,_ and *R*_d_ are as described above.

The *LSP* was calculated by. Formula 3.

The *LCP* can be obtained by:

$$LCP=\frac{\left(R_{d}\times Pmax-\theta \times {Rd}^{2}\right)}{a\times (Pmax-Rd)}$$ (6)

Where: *LCP* represents the light compensation point (μmol (photon) m^-2^·s^-1^); k, α, *P*_max_, *R*_d_ are as described above.

The expressions for the exponential model (EM) [18] was represented to the following form:

$$P_{n}=P_{max}\cdot \left(1-e^{\frac{-\alpha I}{P_{max}}}\right)-R_{d}$$ (7)

Where: *e* indicates the base of natural logarithm, *α*, *I*, *P*_max_, *P*_n_, and *R*_d_ are as described above.

The *LSP* was calculated by. Formula 3,

The *LCP* was calculated by. Formula 8.


$$LCP=\frac{Pmax}{\alpha }\times ln\frac{Pmax}{P_{max}-R_{d}}$$ (8)

The modified rectangular hyperbola model (MRHM) [20, 23, 26] was represented to the following form:

$$P_{n}=\alpha \times \frac{1-\beta I}{1+\gamma I}I-R_{d}$$ (9)

where: *β* and *γ* are adjusting factors. *β* represents the photoinhibition item (dimensionless), *γ* represents the light saturation item (dimensionless), and γ = α/*P*_max_. *α*, *I*, and *R*_d_ are as described above.

The *P*_max_, *LCP* and *LSP* were expressed on the modified rectangular hyperbola model in Eqs 10, 11, and 12, respectively:

$$P_{max}=ɑ{\left(\frac{\sqrt{\beta +\gamma }-\sqrt{\beta }}{\gamma }\right)}^{2}-R_{d}$$ (10)

$$LCP=\frac{R_{d}}{\alpha }$$ (11)

$$LSP=\frac{\sqrt{(\beta +\gamma )/\beta }-1}{\gamma }$$ (12)

where *LCP*, *LSP*, *α*, *β*, *γ*, and *R*_*d*_ are as described above.

### Model assessment and validation

The fitting quality of the different models were assessed by mean square errors (*MSE*), determinants coefficients (*R*^2^), and Akaike information criterion (*AIC*), the best combination with the largest *R*^2^ value and smallest *MSE* and *AIC* value represented the higher fitting accuracy.

Mean square error (*MSE*) was the average of squared forecast errors, it is the specific value of the sum of squared errors to the number of errors.


$$MSE=\frac{1}{n}\sum_{i=1}^{n}{\left(y_{i}-{\hat{y}}_{i}\right)}^{2}$$ (13)

Determinants coefficients (*R*^2^) represents the fitting degree of net photosynthetic rate and light intensity.


$$R^{2}=1-\frac{\sum_{i=1}^{n}{\left(y_{i}-{\hat{y}}_{i}\right)}^{2}}{\sum_{i=1}^{n}{\left(y_{i}-\bar{y_{i}}\right)}^{2}}$$ (14)

Akaike information criterion (*AIC*) is a fined technique based on in-sample fit to estimate the likelihood of a model to predict/estimate the future values.

$$AIC=2k+nln\frac{\sum {\left(y_{i}-{\hat{y}}_{i}\right)}^{2}}{n}$$ (15)

where y_i,_ ŷ_i_ and $\bar{y_{i}}$ in the equations above represented the measured value, the fitted value and the mean of the measured values, respectively n is the number of observations. k is the number of estimated parameters [43].

### Statistical analyses

The measured light-response data are collated and analyzed and expressed as mean ± standard deviation (SD) of three replicates. Statistical analyses of the data were performed using two-way analysis of variance (ANOVA) by GraphPad Prism 8.0 or the SPSS software (version 18.0) and Duncan tests. Parameter values which are significantly different (p<0.05) are indicated by different letters.

## Results

### *P*n in response to *PAR*

**Fig 3:**
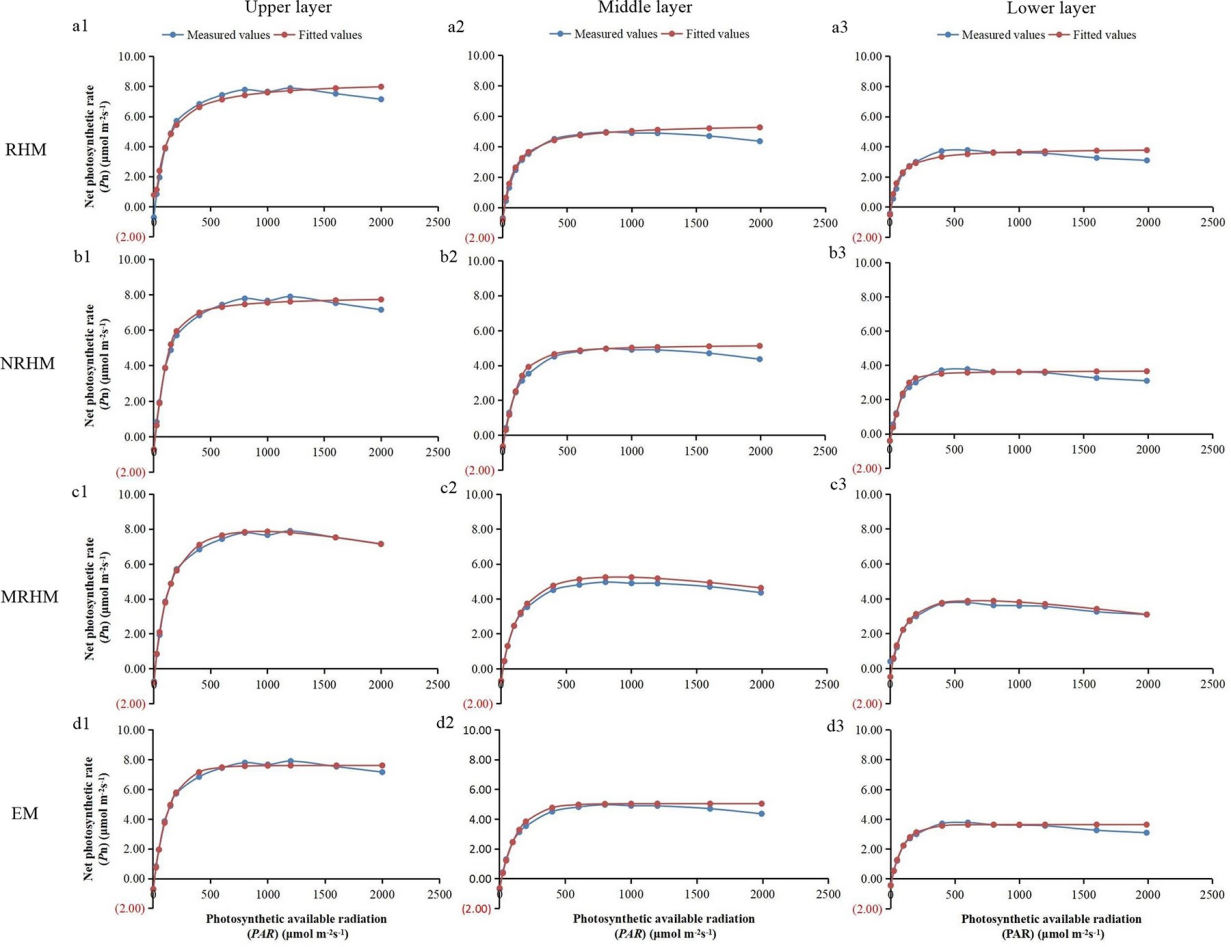
Comparison of the measured values and fitted values of net photosynthetic light response curves in different layers on the RHM (a1-a3), NRHM (b1-b3), MRHE (c1-c3) and EM (d1-d3) models. *PAR* represents photosynthetic available radiation, *MSE* represents mean square error, *R*^2^ represents the coefficient of determination and *AIC* represents Akaike information criterion.

To describe the relationships between *P*_n_ and *PAR*, photosynthetic light-response curves (*P*_n_*-PARs*) of *Larch* was studied. The results showed that light response curves (*P*_n_—*PAR*) fitted by NRHM, EM and MRHM from different layers were of similar tendency, while RHM were difficult to implement because the curves increased gradually with no extreme. Taking the middle layer as an example, the *P*_n_-*PAR* curves could be divided into three stages, the net photosynthetic rate (*P*_n_) increased linearly (rapidly) with the augments of photosynthetic available radiation (*PAR*) in the first stage, where *PAR* < 200 μmol (photon) m^-2^ s^-1^, then increased nonlinearly up to the maximum *P*_n,_ in the second stage, the maximum *P*_n_ is 6.00 μmol (photons) m^-2^s^-1^ when *PAR* is 600 μmol (photons) m^-2^s^-1^, and decreased gradually with increasing *PAR* in the third stage (Fig 3). At the same *PAR*, needles under upper layer had a higher *P*_n_ than those of the middle and lower leaves, and *P*_n_ values under different leaf layer could be ranked as: Upper layer >Middle layer >lower layer.

### Fitting and comparison of photosynthesis-light response curves

The fitting results showed the fitting *P*_n_ values of the four models were very close to the measured values actually when the *PAR* was low (*PAR* <100 μmol (photons) m^-2^s^-1^), the gap increased with an increasing *PAR*, and the difference in *P*_n_ was more remarkable. The *P*_n_ value simulated by NRHM, MRHM, and EM was slightly greater than measured value when *PAR* values are 200–2000 μmol (photons) m^-2^s^-1^, and the simulated pattern of *P*_n_*-PAR* curves showed similar trend, the light-response curve was best described by three models, especially when light intensity (*PAR*) is beyond *P*_max_ (Fig 3). However, the *P*_n_ value simulated by the RHM increased with the increasing *PAR*, the fitting error was too large to use directly (Fig 3A1-3A3). In addition, the mean value of *R*^2^ (range from 0.9748 to 0.9930) of the MRHM model was the highest among the four models, and *MSE* value (*MSE* range from 0.0646 to 0.0866 μmol (photons) m^-2^ s^-1^) and *AIC* value (range from -45.7887 to -34.8321 respective) of the MRHM were significantly smaller than those of other three models in upper, middle and lower layer respectively (Table 2). In addition, MRHM model was superior to other three models in south and north orientation.

**Table 2:** Fitting accuracy of different *P*_n_*-PAR*.

Position	Fitting accuracy	*P*_n_-*PAR* models			
		RHM	NRHM	MRHM	EM
Upper layer	*MSE*	0.2998	0.1903	0.0733	0.129
	*R* ^2^	0.967	0.982	0.993	0.9856
	*AIC*	-14.57	-17.18	-34.8321	-21.1756
Middle layer	*MSE*	0.23	0.1518	0.0646	0.144
	*R* ^2^	0.9628	0.9816	0.9923	0.9746
	*AIC*	-17.06	-20.79	-45.7887	-26.5774
Lower layer	*MSE*	0.4581	0.3	0.0866	0.2398
	*R* ^2^	0.8961	0.9473	0.9748	0.912
	*AIC*	-15.73	-19.27	-37.0383	-22.5198
North	*MSE*	0.3706	0.1988	0.0748	0.1565
	*R* ^2^	0.9563	0.9807	0.9904	0.9735
	*AIC*	-13.71	-17.52	-39.6365	-22.6997
South	*MSE*	0.288	0.2292	0.0748	0.1853
	*R* ^2^	0.9276	0.9598	0.983	0.9413
	*AIC*	-16.32	-20.38	-40.393	-22.943

### Fitting analysis of the photosynthetic parameters based on the models

**Fig 4:**
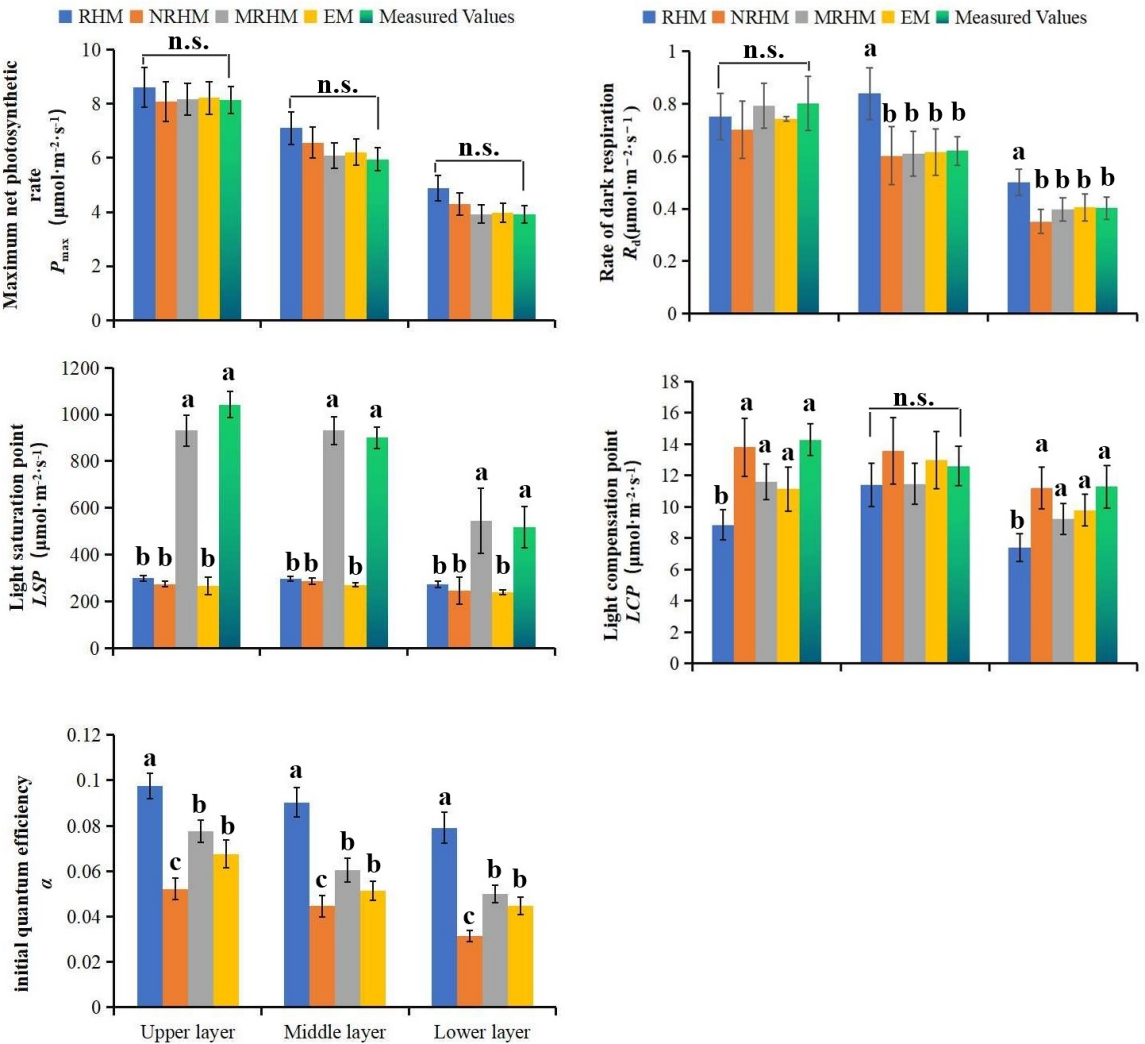
Comparison of light response parameters of different models at the same layer. (a) Parameters of (a) the initial quantum efficiency (*a*). (b) maximum net photosynthetic rate (*P*_max_). (c) light saturation point (*LSP*). (d) light compensation point (*LCP*). (e) dark respiration rate (*R*_d_) for needles at different models. different letters indicate significant difference at p<0.05 level with the least significant difference test, n.s. indicated no significant difference at the level of P<0.05.

The Fitting value of photosynthetic parameters were used to estimate the fitting quality of the model, its accuracy and rationality are affected by the model and layer [16, 19, 20, 27]. Therefore, it is very important to study the fitting effect of different models, different layers and different orientations on light response parameter of needle leaves. The results showed *P*_max_ calculated from the RHM, NRHM and EM were closer to the measured value in the up layer, and was slightly greater than the measured values in the middle and lower layer. The light saturation point (*LSP*) obtained by above three models were much lower than the measured values in the up, middle and lower layer respectively. The fitted values of *P*_max_ and *LSP* by MRHM were close to the measured values in each layer respectively. In addition, the *LSP* values were significant difference between MRHM and the other three models, while the *P*_max_ and *LCP* values were no significant difference, the *a* values of each model, which are the quantum efficiency at low irradiance, ranged from 0 to 0.125 [44], *R*_d_ value was closer among four models (Fig 4).

**Fig 5:**
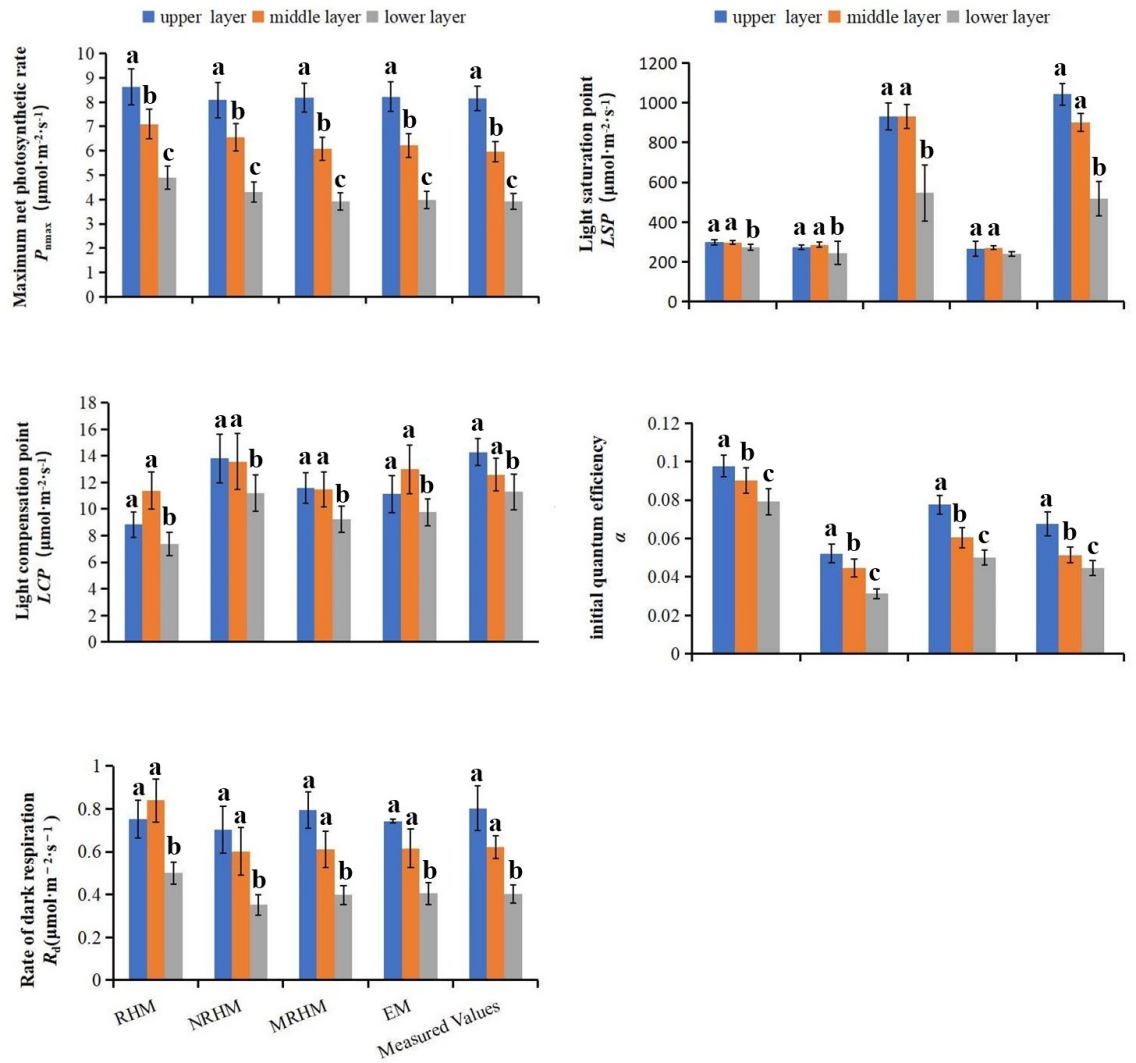
Comparison of light response parameters of different layers at the same model. (a) Parameters of the initial quantum efficiency (*a*). (b) maximum net photosynthetic rate (*P*_max_). (c) light saturation point (*LSP*). (d) light compensation point(*LCP*). (e); dark respiration rate (*R*_d_) for needles at different canyon (different letters indicate significant difference at p<0.05 level with the least significant difference test).

**Fig 6:**
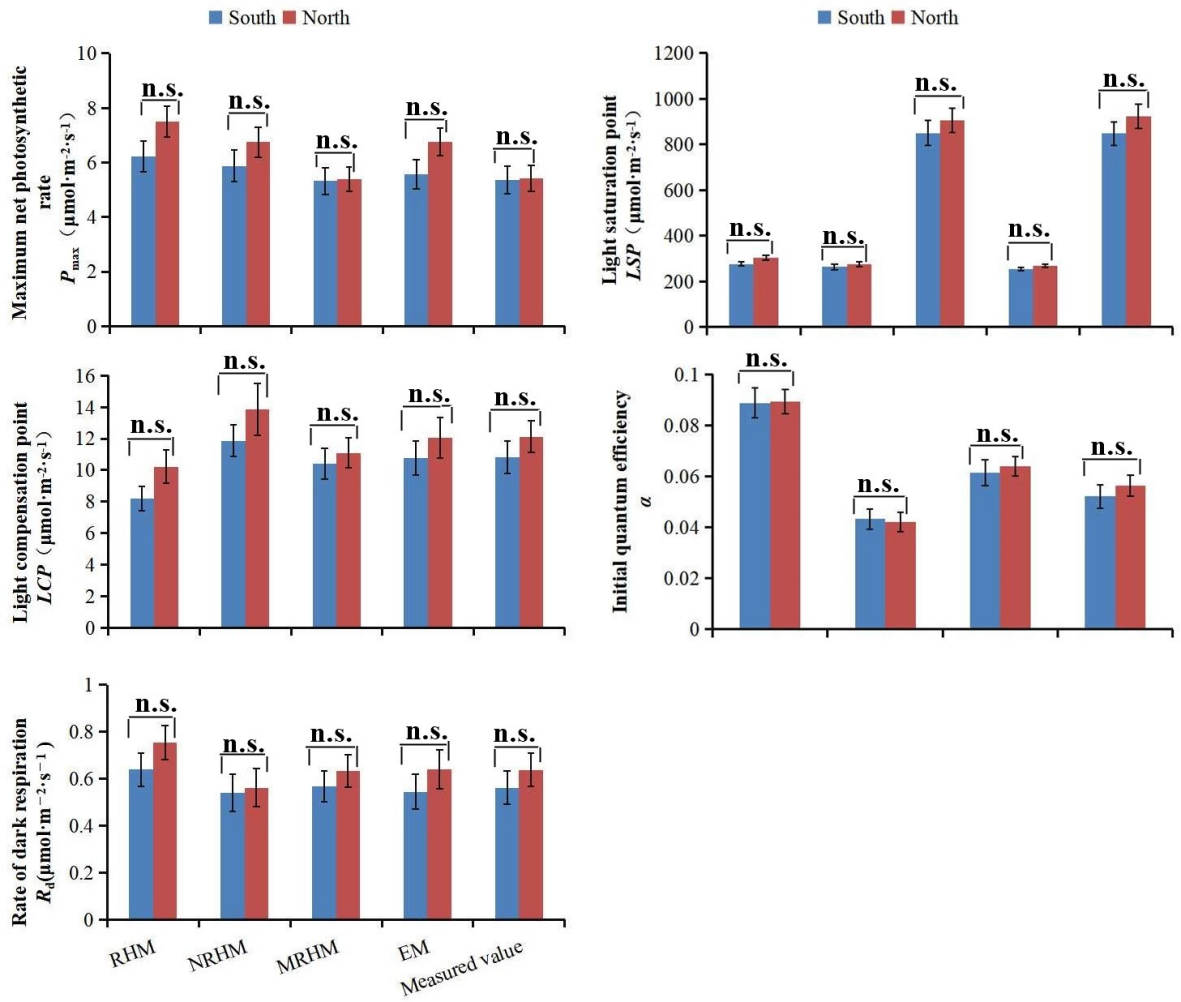
Comparison of light response parameters of different models between south and north direction. (a) maximum net photosynthetic rate (*P*_max_); (b) dark respiration rate (*R*_d_); (c) light saturation point (*LSP*). (d) light compensation point (*LCP*). (e) the initial quantum efficiency (α). (different letters indicate significant difference at p<0.05 level with the least significant difference test).

In the different layers, some simulated values of *P*_n_-*PAR* response parameters revealed somewhat different, there was no significant difference for *LSP LCP* and *R*_d_ in upper and middle layers, but was significant difference in lower layers, the values of *α* and *P*_max_ were significant for in each layer. In addition, the layer is one of the main factors of affecting the light response parameters (e.g. *a*, *P*_max_, *LCP*) (Fig 4, S1 Table). Some simulated values of *P*_n_-*PAR* response parameters calculated by MRHM was more accurate than those obtained from other three models (Fig 5, S1 Table). The photosynthetic parameters which were fitted by four models showed no significant difference between north and south direction (Fig 6, S3 Table).

## Discussion

The light response curve (*P*_n_-*PAR* curve) is an important tool for describing the response of the *P*_n_ to *PAR*, identifying a series of photosynthetic parameters and evaluating the photosynthetic efficiency of plants [11, 45, 46]. Therefore, constructing of *P*_n_-*PAR* curve and choosing the appropriate model are helpful to simulate canopy photosynthesis and predict plant productivity [47, 48]. In the study, the net photosynthetic rate increased initially and then decreased gradually with the increase of *PAR* (Fig 3), which was consistent with the study of leaf *P*_n_-*PAR* curves of some plants in different growth stages [49, 50]. The results indicated light energy absorbed by plants exceeded the needs of plants, the absorption of the excessive light energy would restricted photosynthetic mechanism and series of enzymatic reaction rates in the chloroplasts and result in photo-inhibition of *Larch*. The upper limit value of *P*_n_-*PAR* curve maintained the state of upper layer >middle layer > lower layer during the whole growth period, which was also proved in other *Larch* species [32], indicated that the metabolic capacity was closely related to the light environment [34]. The differences of the *P*_n_-*PAR* curve among different canopies might be associated with leaf characteristics, solar elevation angle, higher chlorophyll a/b ratios, relative depth into crown (RDINC), etc [32, 51]. Additional, The photosynthetic parameters which were fitted by four models showed no significant difference between north and south direction, which was consistent with that of previous study [52].

The fitting of light-response model is an important method to describe the response mechanism of *P*_n_ to *PAR* and evaluate the photosynthetic efficiency [45]. Among four models, the *P*_n_ value simulated by the RHM consistently increased with the increasing *PAR* with no stable or declined trend (Fig 3A1–3A3), indicated that RHM was more suitable for fitting consistently increased type of *P*_n_-*PAR* curves. This result agreed with the previous study [53]. Compared to the other three models, the determinants coefficients (*R*^2^) value of the MRHM was the highest, and mean square errors (*MSE*) value and Akaike information criterion (*AIC*) value were the lowest (Table 2), indicated that MRHM performed better than other three models [23]. In addition, some fitted values of photosynthetic parameters (e.g *P*_max_ and *LSP*) were close to the measured values (Fig 4, S1 Table), Ye [20] has proved that the unique structure of MRHM made it more flexible in simulating different trends of *P*_n_-*PAR* curves.

Compared with data fitted (Fig 3) and error analysis (Table 2) on the *P*_n_-*PAR* curves of the needles at different leaf canopy, there was no significant difference for *LSP*, *LCP* and *R*_d_ in upper and middle layers, but was significant difference in lower layers. However, the values of *a* and *P*_max_ were significant in each layer. The *P*_n_ in the upper canopy was significantly higher than those in the middle and lower canopy, which was not consistent with that of previous study [53]. The different may be due to the comprehensive effects of genetic diversity, different producing areas and environmental factors of trees [54]. It can be seen that layer was one of the main factors of affecting the light response parameters (e.g. *a*, *P*_max_, *LCP*) (Fig 4, S1 Table).

Based on the above discussion, we considered that the light response process and photosynthetic parameter (*P*_max_, *LSP*, *LCP*, and *R*_d_) fitting by the MRHM models was more reliable (S2 Table). and MRHM could fit well the *P*_n_-*PAR* curves of *Larch* (Fig 4, S2 Table). In addition, the *P*_n_-*PAR* of the middle leaf layer can better reflect the changes in leaf layer photosynthetic parameters. However, the results, obtained in the vigorous growth period of *Larix principis rupprechtii* from June to August, are of spatial and temporal limitation in Saihanba, the further studies are needed to better understand the mechanisms of the photosynthetic physiological ecology of plants to the environment.

## Conclusion

This study describe canopy photosynthesis for *Larix principis rupprechtii* plantation, the *P*_n_-*PAR* curves and photosynthetic response parameters were measured under different layer of three planted *L*. *principis-rupprechtii* trees by different models during the growing season. The results showed that the fitting effect of MRHM model was superior to those of other three models and it could analyze the light-response data more accurately, the selection of the middle layer of the plant is the best when measuring the photosynthetic performance of the whole tree in combining with the analysis of fitting precision (Fig 3), the accurate values were as follows: α, *P*_max_, *LSP*, *LCP* and *R*_d_ were 0.06, 6.08 μmol·m^-2^s^-1^, 931.08 μmol·m^-2^s^-1^, 11.45 μmol·m^-2^s^-1^ and 0.61 μmol·m^-2^s^-1^, respectively. This study not only helps to further explore the spatial heterogeneity of carbon sequestration capacity of *Larix principis rupprechtii* leaves in canopy, but also provides a scientific and effective guidance for accurately estimating the productivity of *Larix principis rupprechtii* plantation.

## Supporting information

S1 Table *P*_n_-*PAR* response parameters of leaves in different layers of *Larch*. Black letter ‘a’ indicates significant differences between models, red letter ‘a’ indicates significant differences among different layers.
(DOC)

S2 Table Analysis of light response parameters at different layers. (DOC)

S3 Table Analysis of light response parameters between south and north. (DOC)
