# Supplementary material for: Effects of photosynthetic models on the calculation results of photosynthetic response parameters in young Larix principis-rupprechtii Mayr. plantation
Source: PLoS One. 2021 Dec 31;16(12):e0261683. doi: 10.1371/journal.pone.0261683 (PMC8722371; doi:10.1371/journal.pone.0261683)
Supplement: S1 Table — Black letter ‘a’ indicates significant differences between models, red letter ‘a’ indicates significant differences among different layers. (DOC) [file pone.0261683.s001.doc]

S1 Table *P*_n_-*PAR* response parameters of leaves in different layers of *Larch*.

| Position | models | photosynthetic response parameters | | | | |
| --- | --- | --- | --- | --- | --- | --- |
|  |  | α | *P*_max_ （μmol·m^-2^·s^-1^） | *LSP*  （μmol·m^-2^·s^-1^） | *LCP*  （μmol·m^-2^·s^-1^） | *R*_d_  （μmol·m^-2^·s^-1^） |
| Upper layer | RHM | 0.0976±0.0056aa | 8.6213±0.7412aa | 298.1227±12.5239aa | 8.8384±0.9581aa | 0.7519±0.0885aa |
|  | NRHM | 0.0522±0.0048ba | 8.088±0.7275aa | 273.9457±11.9378aa | 13.8023±1.8477ba | 0.7020±0.1092aa |
|  | MRHM | 0.0775±0.0049ca | 8.1739±0.5859aa | 930.7956±66.8574ba | 11.5768±1.1400ba | 0.7936±0.0854aa |
|  | EM | 0.0675±0.0062ca | 8.2217±0.6033aa | 266.7832±37.5695aa | 11.1319±1.3932ba | 0.7429±0.0085aa |
|  | measured value | --- | 8.1514±0.5024aa | 1041.9365±55.512ba | 11.2532±1.0421ba | 0.8024±0.1235aa |
| Middle layer | RHM | 0.0903±0.0066ab | 7.1015±0.5958ab | 297.6017±10.5085aa | 11.3901±1.3726aa | 0.8389±0.0996aa |
|  | NRHM | 0.0446±0.0047bb | 6.5577±0.5730ab | 285.9184±13.0705aa | 13.5611±2.1041ba | 0.6019±0.1110aa |
|  | MRHM | 0.0605±0.0053cb | 6.0812±0.4623ab | 931.0838±58.4459ba | 11.4542±1.3175ba | 0.6104±0.0853aa |
|  | EM | 0.0514±0.0042cb | 6.2183±0.4906ab | 270.7322±9.5470aa | 12.9861±1.8185ba | 0.6161±0.0897aa |
|  | measured values | --- | 5.9541±0.4135ab | 900.1666±45.426ba | 11.8234±1.2136ba | 0.6213±0.0546aa |
| Lower layer | RHM | 0.0791±0.0068ac | 4.8858±0.4701ac | 273.1065±14.7001ab | 7.3925±0.8819ab | 0.4998±0.0508ab |
|  | NRHM | 0.0313±0.0025bc | 4.3024±0.4080ac | 245.0856±58.4587ab | 11.1963±1.3472bb | 0.3514±0.0468bb |
|  | MRHM | 0.0500±0.0038cc | 3.9262±0.3507ac | 546.1658±139.5438bb | 9.2353±0.9835bb | 0.3985±0.0443bb |
|  | EM | 0.0447±0.0038cc | 3.9840±0.3528ac | 239.3889±9.9733ab | 9.7741±1.0132bb | 0.4056±0.0512bb |
|  | measured values | --- | 3.9176±0.3312ac | 516.9583±87.456bb | 9.2521±0.8346bb | 0.4121±0.0425bb |

Note: Black letter ‘a’ indicates significant differences between models, red letter ‘a’ indicates significant differences among different layers.
