# Supplementary material for: Effects of photosynthetic models on the calculation results of photosynthetic response parameters in young Larix principis-rupprechtii Mayr. plantation
Source: PLoS One. 2021 Dec 31;16(12):e0261683. doi: 10.1371/journal.pone.0261683 (PMC8722371; doi:10.1371/journal.pone.0261683)
Supplement: S2 Table — (DOC) [file pone.0261683.s002.doc]

S2 Table Analysis of light response parameters at different layers

| parameter | Upper layer | Middle layer | lower layer |
| --- | --- | --- | --- |
| α | 0.0775±0.0049a | 0.0605±0.0053b | 0.0500±0.0038c |
| *P*_max_ | 8.1739±0.5859a | 6.0812±0.4623b | 3.9262±0.3507c |
| *LSP* | 930.7956±66.8574a | 931.0838±58.4459a | 546.1658±139.5438b |
| *LCP* | 11.5768±1.1400a | 11.4542±1.3175a | 9.2353±0.9835b |
| *R*_d_ | 0.7936±0.0854a | 0.6104±0.0853a | 0.3985±0.0443b |
