# Supplementary material for: Effects of photosynthetic models on the calculation results of photosynthetic response parameters in young Larix principis-rupprechtii Mayr. plantation
Source: PLoS One. 2021 Dec 31;16(12):e0261683. doi: 10.1371/journal.pone.0261683 (PMC8722371; doi:10.1371/journal.pone.0261683)
Supplement: S3 Table — (DOC) [file pone.0261683.s003.doc]

S3 Table Analysis of light response parameters between south and north

| Position | models | photosynthetic response parameters | | | | |
| --- | --- | --- | --- | --- | --- | --- |
|  |  | α | *P*_max_ （μmol·m^-2^·s^-1^） | *LSP*  （μmol·m^-2^·s^-1^） | *LCP*  （μmol·m^-2^·s^-1^） | *R*_d_  （μmol·m^-2^·s^-1^） |
| South | RHM | 0.0888±0.0058 | 6.2302±0.5688 | 276.41355±9.9147 | 8.192±0.7868 | 0.6401±0.0707 |
|  | NRHM | 0.0433±0.0039 | 5.8804±0.5649 | 262.4386±11.6296 | 11.8618±1.0168 | 0.5411±0.0804 |
|  | MRHM | 0.0615±0.005 | 5.321±0.4937 | 850.0696±54.4774 | 10.4164±0.9644 | 0.5681±0.0656 |
|  | EM | 0.0521±0.0047 | 5.5582±0.5365 | 253.8549±8.334 | 10.78±1.0818 | 0.5451±0.0744 |
|  | measured value | --- | 5.3574±0.4985 | 848.1296±51.1562 | 10.8254±1.0234 | 0.5623±0.0712 |
| North | RHM | 0.0892±0.0048 | 7.5088±0.5627 | 302.8071±10.4633 | 10.2218±1.0354 | 0.7537±0.0721 |
|  | NRHM | 0.0421±0.0037 | 6.7519±0.5466 | 274.1945±10.1997 | 13.8447±1.661 | 0.5624±0.082 |
|  | MRHM | 0.0639±0.0038 | 5.3999±0.4503 | 905.2939±54.3621 | 11.0945±0.946 | 0.6335±0.0696 |
|  | EM | 0.0564±0.0041 | 6.7619±0.5007 | 266.6128±7.346 | 12.0594±1.2962 | 0.6411±0.0825 |
|  | measured values | --- | 5.4212±0.4856 | 923.3652±52.2894 | 12.1245±0.9982 | 0.6385±0.0706 |
